# A study protocol for a European, mixed methods, prospective, cohort study of the effectiveness of naloxone administration by community members, in reversing opioid overdose: NalPORS

**DOI:** 10.1186/s12889-023-16445-6

**Published:** 2023-08-24

**Authors:** Nicola Metrebian, Ben Carter, Desiree Eide, Rebecca McDonald, Joanne Neale, Stephen Parkin, Teodora Dascal, Clare Mackie, Ed Day, Joar Guterstam, Kirsten Horsburgh, Martin Kåberg, Mike Kelleher, Josie Smith, Henrik Thiesen, John Strang

**Affiliations:** 1 National Addiction Centre, King’s College London, London, UK; 2 Biostatistics and Health Informatics, King’s College London, London, UK; 3 Norwegian Centre for Addiction Research, University of Oslo, Oslo, Norway; 4 Birmingham & Solihull Mental Health NHS Foundation Trust, Birmingham, UK; 5 Department of Clinical Neuroscience, Centre for Psychiatry Research, Karolinska Institutet, & Stockholm Health Care Services, Stockholm County Council, Stockholm, Sweden; 6 Scottish Drugs Forum, Glasgow, Scotland, UK; 7 Stockholm Centre for Dependency Disorders, Stockholm, Sweden; 8 Department of Global Public Health, Karolinska Institutet, Stockholm, Sweden; 9 South London and Maudsley NHS Foundation Trust, London, UK; 10 Public Health Wales, Cardiff, Wales UK; 11 Sundheds Team, City of Copenhagen, Denmark

**Keywords:** Opioid overdose, Naloxone, Take home naloxone, Heroin, Emergency, Resuscitation, Mortality, Death

## Abstract

**Background:**

Worldwide, opioid use causes more than 100,000 overdose deaths annually. Naloxone has proven efficacy in reversing opioid overdoses and is approved as an emergency antidote to opioid overdose. Take home naloxone (THN) programmes have been introduced to provide ‘community members’, who are likely to observe opioid overdoses, with naloxone kits and train them to recognise an overdose and administer naloxone. The acceptability and feasibility of THN programmes has been demonstrated, but the real-life effectiveness of naloxone administration by community members is not known. In recent years, the approval of several concentrated naloxone nasal-spray formulations (in addition to injectable formulations, eg.prenoxad) potentially increases acceptability and scope for wider provision. This study aims to determine the effectiveness of THN (all formulations) in real-world conditions.

**Methods:**

A European, multi-country, prospective cohort study, to assess the use of THN by community members to reverse opioid overdoses in a six-month, follow-up period. Participants provided with THN from participating harm reduction and drug treatment sites will be recruited to the study and followed-up for six months. We are particularly interested in the experiences of community members who have been provided with THN and have witnessed an opioid overdose. All participants who witness an opioid overdose during the six-month period (target approx. 600) will be asked to take part in a structured interview about this event. Of these, 60 will be invited to participate in a qualitative interview. A Post Authorisation Efficacy Study (PAES) for the concentrated nasal naloxone, Nyxoid, has been integrated into the study design.

**Discussion:**

There are many challenges involved in evaluating the real-life effectiveness of THN. It is not possible to use a randomised trial design, recruitment of community members provided with THN will depend upon recruitment sites distributing THN kits, and the type of THN received by participants will depend on regulations and on local clinical and policy decision-makers. Following up this population, some of whom may be itinerant, over the 6-month study period will be challenging, but we plan to maintain contact with participants through regular text message reminders and staff contact.

**Trial registration:**

ClinicalTrials.gov Identifier: NCT05072249. Date of Registration: 8.10.2021

**Supplementary Information:**

The online version contains supplementary material available at 10.1186/s12889-023-16445-6.

## Background

Opioid use is a global public health issue, causing more than 100,000 overdose deaths annually [1–4]. In Europe, 5,141 overdose deaths involving illicit drugs were reported in 2019, primarily from opioids [5]. In 2019, the mortality rate due to overdose was estimated at 14.8 deaths per million of the adult population in Europe [5].

Naloxone is a µ-opioid receptor antagonist which can reverse the respiratory depression effects of opioids by blocking receptors. It is approved as a clinical antidote to opioid overdose [6, 7]. Naloxone rapidly reverses the acute effects of opioids (e.g., heroin, fentanyl, methadone, buprenorphine, codeine, morphine, tramadol, and oxycodone) including extreme drowsiness, slowed breathing, and loss of consciousness, thereby having ability to prevent opioid overdose fatality.

Naloxone has been used since the 1970s to treat opioid overdose in hospital and pre-hospital settings [1, 8]. It has proven to be safe and effective when administered in time and at the right dose [7, 9]. Since the late 1990s, take home naloxone (THN) programmes have been introduced [10, 11]. Such programmes provide ‘community members’ who are likely to observe opioid overdoses (including people who use opioids themselves, their family, and carers) with naloxone kits and train them to recognise an overdose, call an ambulance, administer naloxone and perform basic life support [1].

Naloxone has proven efficacy and can be administered by the intravenous (IV), intramuscular (IM), subcutaneous (SC) or intranasal (IN) routes [7, 12–14] and the acceptability and feasibility of THN programmes have been demonstrated in many contexts [15–18]. A systematic review applying the Bradford Hill criteria to findings from existing non-randomized studies, found that THN programmes have led to improved survival rates among programme participants and reduced heroin overdose mortality rates in the community [19]. However, the real-life effectiveness of naloxone administration by community members in reversing opioid overdose is unknown. Conducting a randomised controlled trial in this setting would not be ethical. This study will be undertaken at a time of change for THN programmes in Europe, as new concentrated naloxone nasal-spray formulations have become available, sometimes alongside pre-filled syringes of injectable naloxone which have previously been offered through these programmes. All types of THN formulations will be examined in this study.^1^

This study aims to determine the effectiveness of THN in real world conditions. Study objectives include, assessing the rate of administration of naloxone and subsequent reversal of opioid overdose by community members who witness an opioid overdose, determining how closely real-life use conforms to overdose response training and obtaining a better understanding of naloxone administration and related overdose reversals by community members.

## Methods/design

### Study design and setting

This is a mixed-method, prospective, cohort study, to assess the effectiveness of naloxone administration and overdose reversal by community members across a number of European countries.

Quantitative data collection alone in the form of structured interviews cannot adequately capture the many interconnecting social, economic, and cultural factors that influence response to opioid overdose as well as the outcomes of such intervention. Therefore, a mixed methods approach, incorporating qualitative interview data, was selected for this study. The protocol was designed after discussions with people who use heroin and other opioids and who have been provided with take home naloxone. We consulted with various communities of service users with living and lived experience, including patients in opioid treatment, individuals currently using opioids and at risk of overdose, individuals in recovery, and commissioners and provider organisations. They told us that overdose is of major concern, that they would be happy to answer questions about their experience of witnessing an overdose and administering naloxone (or not) and advised on the timings and content of the interviews.

Due to COVID-19 social distancing restrictions before July 2021, the study includes methods that allow for enrolment and follow-up interviews to be carried out remotely.

**Fig. 1:**
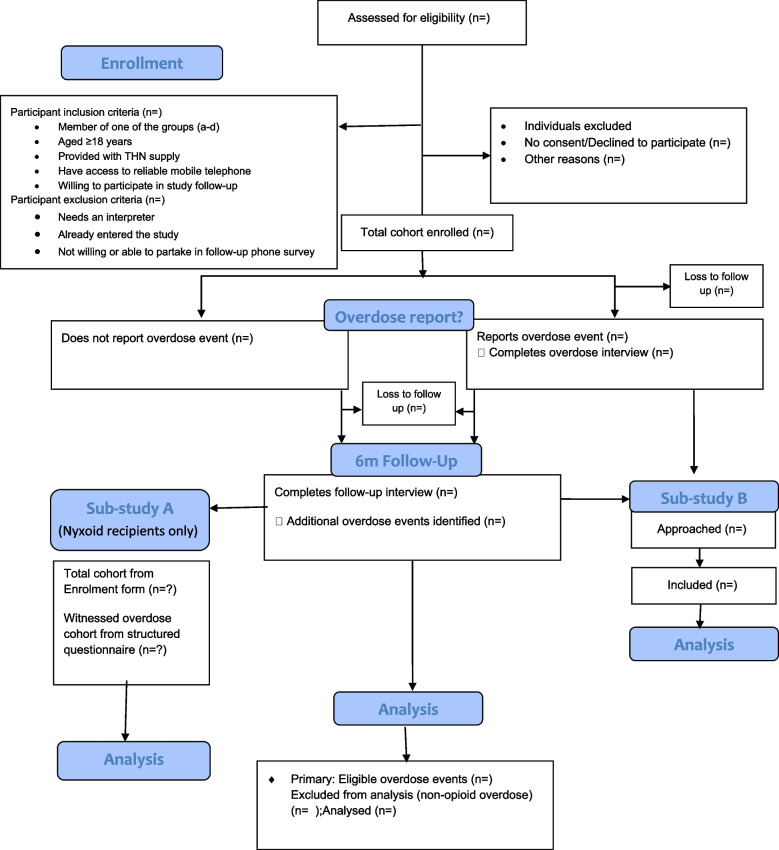
Consort flow diagram

Participants, provided with THN from participating harm reduction and drug treatment sites, will be recruited and followed-up for six-months (Total cohort). All participants who witness an opioid overdose during the six-month period will be asked to participate in a structured interview about this event (Witnessed overdose cohort). In addition, there are two nested sub-studies. Sub-study A comprises two structured surveys, to assess the concentrated naloxone nasal-spray formulation training materials. Sub-study B comprises qualitative interviews conducted with a sample of those who have witnessed an opioid overdose and undertaken a structured interview to better understand the use, safety, and effectiveness of different naloxone products (Fig. 1). A Post Authorisation Efficacy Study (PAES) for the concentrated nasal naloxone, Nyxoid, has been integrated into the study design.

Conducting the study in multiple European Economic Area (EEA) countries will increase the generalisability of findings across Europe. There are various medical, treatment, cultural and health care system related factors and drug use and treatment approaches which differ across European countries. Due to these factors, the introduction of naloxone programmes, and the forms of naloxone provided (e.g., concentrated naloxone nasal spray or pre-filled syringes of injectable naloxone) are not evenly distributed over EEA countries.

The study will be undertaken across several European countries. Participating countries will be chosen based on, whether they report high rates of opioid use and fatal overdoses, and provision of THN: they are thus likely to be countries with the highest rates of drug-induced mortality in Europe [5]. We anticipate that they will provide an approximate estimated equal split between those providing nasal and injectable forms of naloxone.

Countries need to be able to recruit the target number of participants and for such participants to be contactable for follow-up (particularly the expected 10% who will witness an opioid overdose within the study period).

Within each country, recruitment sites will need to fulfil the following criteria:

- routinely provide THN
- be an active drug treatment facility, needle and syringe programmes (NSP) or community/family support group for people who use opioids
- can reasonably commit to recruit a substantial number of people that (combined with the other sites in the country) can reach the country target of 1,000 participants

### Study population

Individuals provided with THN from participating harm reduction and treatment sites in each of the countries will be recruited to the study. Participants receiving THN (reflecting usual clinical practice of providing THN to community members who are likely to observe an opioid overdose) will include the following groups:

- individuals with an opioid use disorder and in contact with treatment services
- individuals with an opioid use disorder and out of contact with treatment services, but in contact with harm reduction services (e.g., NSP schemes, supervised consumption facilities and outreach)
- family members and carers of individuals with an opioid use disorder
- health /social care staff (not including medically trained health care professionals)

We do not seek to influence the prescribing activity of the services, and so we are not seeking to influence the proportions in each group to whom THN is provided. However, based on observations of recent clinical practice, we consider that it is likely that participants will be recruited mostly from eligibility groups a) and b).

Inclusion criteria will include:

- Member of one of the groups above (a-d)
- Aged ≥ 18 years
- Provided with THN supply at time of enrolment (or can demonstrate that they have a supply of THN (in-date) and have received training at time of enrolment)
- Have access to reliable mobile telephone and can present it at the point of enrolment
- Willing to participate in study follow-up
- Provided verbal or written informed consent

Exclusion criteria will include:

- Needs an interpreter
- Already entered the study
- Not willing or able to partake in follow-up phone survey

Between June 2021 and April 2024, we intend to recruit the total cohort of 6,000 participants, including approx. 3,000 participants supplied with concentrated naloxone nasal spray and 3,000 with pre-filled syringes of injectable naloxone. With each country recruiting approximately 1,000 participants.

However, our main interest is in recruiting our witnessed overdose cohort of approximately 600 participants, including approximately 300 provided with the concentrated naloxone nasal-spray. These study participants will include those who have witnessed an opioid overdose in the 6-month study period (and completed a structured interview). Of these, 60 participants from England, Scotland, or Wales, will also participate in a longer, in-depth qualitative interview conducted by telephone (Sub-study B). We focus on collecting data on one witnessed overdose per participant.

### Objectives and outcome

#### Main study objectives

There are two co-primary objectives:

1. To determine the frequency of deaths in the 24 h (or later if information is available) subsequent to administration of naloxone by community members, to reverse an opioid overdose in real world settings.
2. To determine the proportion of naloxone administration with the intention of reversing an opioid overdose by community members provided with THN who witness an opioid overdose.

Secondary objectives include:

a Daily carriage rate of THN amongst community members supplied with THN over a 6-month period
b Proportion of community members supplied with THN who witness an opioid overdose over a 6- month period
c Proportion of community members supplied with THN who witness an opioid overdose, where there is THN present
d Proportion of community members supplied with THN, who witness an opioid overdose, where there is THN present and who administer THN to reverse an opioid overdose
e Proportion of community members who report a recurrence of respiratory depression in a person who has been administered THN for an opioid overdose within 1-h of THN administration
f Proportion of community members who report a second dose of naloxone administered to a person experiencing an opioid overdose within 1-h of administration of the first dose of THN
g Proportion of community members who report opioid withdrawal symptoms and/or angry (including abusive or hostile) reaction precipitated when THN is administered for the reversal of an opioid overdose
h Survival rate of individuals having received naloxone at 2 h post—administration of THN or at arrival of ambulance/medical assistance if this occurs before 2 h have passed since the THN was administered, as far as this can be ascertained
i Proportion of fatal outcomes within a 12-h period after the witnessed opioid overdose, as far as this can be ascertained
j In case of fatal outcome: what is known about the cause of the fatal outcome (other than fatal overdose), based on any direct or second-hand information available to the participant (e.g., delays in delivery of intervention such that death or irreversible brain damage already occurred, and/or failures by the responder such as failure to call emergency services or failure to administer naloxone, and/or failure of the naloxone to reverse the overdose, and/or failure of the medication device)
k Accuracy of diagnosis of a probable opioid overdose by community member
l Appropriateness of response to opioid overdose by community member
m Explore the use, safety, and effectiveness of different naloxone products
n Percentage of participants, who are at a risk of experiencing an opioid overdose themselves, who report experiencing an opioid overdose (fatal or non-fatal) during the study period

#### Sub-study A

Objectives include the extent to which the participant read, understood, and followed the concentrated naloxone nasal-spray instructions.

#### Sub-study B

Objectives include, providing more detailed insights into a witnessed overdose situation.

#### Main study outcomes

Co-primary outcomes (of witnessed overdose cohort) include:

Among community members provided with a supply of THN and witnessing an opioid overdose, a) the proportion administering THN to reverse an opioid overdose, and the frequency of deaths within 24-h (or later if information is available) after administration of THN. Overdose events eligible for inclusion in the primary outcome analysis are opioid overdoses witnessed by the participant during the six-month period after study enrolment.

Secondary outcomes include:

Of the Total cohort

- Percentage of participants who report carrying THN (on day of interview)
- Percentage of participants who witness an opioid overdose over 6-month period
- Percentage of participants, who are at a risk of experiencing an opioid overdose themselves, who report experiencing an opioid overdose (fatal or non-fatal)”

Of the Witnessed overdose cohort:

- Percentage of participants who are carrying THN
- Percentage of participants who report there was THN present
- Percentage of participants who state there was THN present, and administer THN
- Percentage of participants who administer THN and report that respiratory depression occurred in the person they resuscitated within one hour of THN administration
- Percentage of participants who report administering a second dose of THN to a person experiencing an opioid overdose, within 1-h post administration of first dose of THN
- Percentage of participants who report that withdrawal symptoms and/ or anger, rage or violence occurred in person they resuscitated with THN. Frequency of each symptom witnessed (at most recent overdose witnessed)
- Percentage of participants who report that the person receiving naloxone has survived at 2 h post administration of naloxone, OR at arrival of ambulance/medical assistance, if this occurs before 2 h have passed since the opioid overdose
- Percentage of participants who report fatal outcomes within 2 h of the identification of opioid overdose, or at the arrival of an ambulance/medical assistance, where THN was administered and where THN was not administered
- Percentage of participants who correctly diagnose opioid overdose crisis (assessed through multiple response questions)

Data on wider response to the overdose, in addition to naloxone, will be collected. This will include: tried to wake them verbally (shouted/ called the person’s name), tilted head back, checked for response, checked breathing, put into recovery position, chest compressions, rescue breathing / mouth-to-mouth, cleared airways, checked pulse, ambulance called, ambulance gave naloxone.

Data will be captured through a structured questionnaire developed specifically for this study (See Supplementary information: Questionnaire). The questionnaire was informed by literature reviews, clinical guidelines, previous research undertaken by the authors, and consultations with service users with living and lived experience. It was piloted before the study started. The main observation window has been limited to one hour because, death from an opioid overdose is likely to be within minutes to hours and medical care is expected to have been handed over to medical practitioners (e.g., paramedics, ambulance personnel, etc.) within this time-period. The potential resuscitating effects of naloxone are also effective within a short period (minutes) and of limited duration (minutes to hours; assuming that naloxone has been administered at a time when no irreversible damage has occurred). Deaths occurring outside of the 1-h time window may be indirectly related to the overdose (e.g., aspiration pneumonia, anoxic brain trauma, multiorgan failure due to rhabdomyolysis) and because of either zero or late interventions. They would not be caused by a failure of naloxone to acutely treat the respiratory depression due to opioid overdose. Moreover, participants may not know the opioid overdose patient sufficiently and may be unlikely to be able to provide longer term follow-up information. However, because of possible delays in pronouncing death, a 2-h time limit will be used as far as it can be ascertained.

*Sub-study A outcomes:*

Of the Total cohort:

- Percentage of participants who received various forms of training materials during their naloxone training

Of the Witnessed overdose cohort:

- Percentage of participants who appropriately recognised and responded to an overdose

*Sub-study B outcomes:*

- Increased understanding of carriage of naloxone products and disposal of used kits
- Increased understanding of how naloxone is administered in an emergency overdose situation, including any problems encountered (e.g., device malfunction or misuse)
- Increased understanding of decision-making processes relating to naloxone administration in an emergency opioid overdose situation, including giving/not giving a second dose
- Increased understanding of the impact of the social and environmental setting in which the overdose occurs – on naloxone use
- Increased understanding of the links between naloxone carriage and use on willingness to call an ambulance when witnessing an opioid overdose
- Increased understanding of the effectiveness of naloxone administration, including speed of reversal and opioid overdose outcome
- Increased understanding of any negative consequences of naloxone administration (e.g. withdrawals, aggression, respiratory depression and death by the person who experienced the overdose) and their implications for future opioid overdose events witnessed
- Increased understanding of care of the person who experienced the overdose both during the opioid overdose and post-naloxone administration
- Increased understanding of knowledge of naloxone, including training and information needs
- Increased understanding of views on the THN training materials

In addition, the clinician assessment will establish:

- Accurate diagnosis of opioid overdose (5-point Likert scale) through expert assessment of transcripts
- Accurate identification of respiratory depression (5-point Likert scale), through expert assessment of transcripts
- Appropriate response to opioid overdose (5-point Likert scale) through expert assessment of transcripts
- Adequate post naloxone aftercare (5-point Likert scale) through expert assessment of transcripts

### Study procedures

#### Recruitment of participants

Individuals to whom THN has been supplied will be screened for eligibility for the study. If eligible, they will be provided with information about the study and asked whether they are willing to participate. Additional consent will be sought from participants who use drugs (groups a. and b.) to use their personal data for linkage to the national/regional death registers to identify any deaths during the study period. Consenting participants will be required to send a text message to the study’s automated telephone system, containing their unique study number (PIN) and initials to be formally enrolled into the study. These data will be captured on the system with a date stamp. They will receive a message by return, welcoming them to the study and this prompts the system to send monthly reminder texts to their mobile.

Staff at recruitment sites can recruit participants face to face or remotely by telephone.

##### Enrolment

All consenting participants will be asked to complete a baseline/enrolment questionnaire with the member of staff recruiting them into the study. The questionnaire will elicit baseline information on sociodemographic characteristics, previous drug use, previous experience of witnessed or experienced overdoses, naloxone provision and training. Participants will be reimbursed for their time with a £5 shopping voucher. In addition, the staff member will complete a brief record of the naloxone and training provided to the participant at the time of recruitment to the study.

#### Follow-up assessments

##### Total cohort

Participants will be sent monthly text message reminders over the 6-month study period to prompt them to contact research staff if they have witnessed an opioid overdose and take part in a witnessed overdose interview. By interviewing participants as soon as possible after they have witnessed an overdose, we hope to reduce any recall issues. Staff who have frequent or regular contact with participants (including instances of replenishment of naloxone supply) will also prompt participants to contact research staff to notify them of any witnessed opioid overdose.

All participants will be contacted by telephone at six months by the research staff and invited to take part in a structured follow-up interview. The interview ascertains if the participant has witnessed an overdose since enrolment (or since contacting the research staff if relevant), actions and outcomes relating to the overdose and use of THN, carriage of THN, and elements relating to their overdose prevention/THN training. All participants who complete this interview will be reimbursed with a £15 shopping voucher.

To reduce loss to follow-up, we will collect contact details from participants at enrolment and provide participants with a modest monetary reimbursement for undertaking the structured interview at six months and the witnessed overdose interview.

##### Witnessed overdose cohort

If the participant reports that they have witnessed an overdose, they will be contacted by research staff and asked to complete a structured questionnaire administered by the researcher either face to face or over the telephone. This questionnaire covers details relating to the overdose event and use of THN, THN carriage, and elements relating to their overdose prevention/THN training. Participants will be reimbursed with a £15 shopping voucher for their time.

##### Sub-study A

The aim of sub-study A is to better understand the effectiveness of the concentrated naloxone nasal-spray educational and training materials delivered to participants. Sub-study A comprises two structured surveys, embedded in i) the Training Form undertaken at Baseline/Enrolment, and ii) the structured questionnaire administered to those participants who report they have witnessed an opioid overdose. Survey one examines training and materials provided and Survey two asks participants about the extent to which they followed the instructions (or equivalent).

##### Sub-study B

The aim of sub-study B is to better understand the use, safety, and effectiveness of different naloxone products. Sub-study B comprises interviews with 60 UK participants from the witnessed overdose cohort. Between 20–40 of these 60 participants will have been provided with concentrated naloxone nasal-spray, while the remainder will have been provided with pre-filled syringes of injectable naloxone.

Qualitative interviews will be conducted by telephone, by a trained researcher. Each interview will be led by a topic guide, last approximately one hour, and be audio recorded. The topic guide has been developed by the authors JN and SP (see Supplementary information: Topic guide). The topic guide will briefly cover the participant’s personal circumstances, current substance use, and treatment situation. It will then capture details of any overdoses experienced or witnessed since the study started before focusing in more depth on the most recent overdose witnessed (circumstances of the opioid overdose, opioid overdose onset, response to the opioid overdose, naloxone administration, impact of the social and environmental setting on naloxone carriage rate and use, effectiveness of naloxone, outcome of the opioid overdose, and longer-term impact of the opioid overdose on the participant). Participants will also be asked about any training they have had, or need, in naloxone administration.

In addition, after the interview, an independent clinical adviser/consultant will review the information and assess the accuracy of the participant’s identification and response to the opioid overdose (as detailed in the transcribed interviews) using a pre-designed 5-point Likert Scale.

#### Mortality data

In addition, the national/regional death registers will be probed to confirm mortality data for study participants who are at risk of experiencing an opioid overdose themselves. Death records will be checked for opioid overdose as cause of death, including toxicology information where available.

#### Safety

Serious and non-serious adverse drug reactions (SADR and ADR) suspected to have a causal relationship with the administration of take-home naloxone will be captured.

Collecting and monitoring safety data in this study is challenging. We are examining the administration of naloxone for overdose reversal, by community members, in a real world setting. Given the nature of this study, serious adverse drug reactions (SADRs) and adverse drug reactions (ADRs) observed by the study participant witnessing the overdose will be reported. ADRs will be captured retrospectively during the follow-up research interviews. This is considered a reliable source of data by the funder and manufacturer of the concentrated nasal naloxone, nyxoid who require this safety data.

As complete, as possible, narrative information on the suspected ADR and its outcome will be collected. However, we recognise that obtaining follow-up information on ADRs may be difficult within this study setting.

Participants witnessing an opioid overdose will specifically be asked about SADR and ADRs in follow-up interviews, and record answers. Fatal outcomes of overdoses are one of the endpoints of the study and will not be reported as SADRs, except if the fatal outcome is considered to have been related to THN administration or due to lack of efficacy of THN administration.

In addition, to each country's standard responsibilities on clinicians to report any adverse drug reactions to naloxone, any serious adverse drug reactions related to the concentrated naloxone nasal-spray will be reported to the manufacturer’s Drug Safety Team within 2 calendar days counting from first day of awareness (Day 0). Non-serious adverse drug reactions suspected to have a causal relationship with the concentrated naloxone nasal-spray will be reported monthly to the manufacturer’s Drug Safety Team.

### Study analysis

The rate of administration of THN by community members witnessing an opioid overdose and the frequency of deaths in the 24 h (or later if information is available) after administration of naloxone will be estimated as proportions. The study data will be stratified by participant groups as follows: 1) Patients in treatment for opioid use disorder (OUD), 2) Participants who use opioids but are not currently in treatment, 3) Friends and family members, 4) Staff working with individuals with OUD. Effective administration rates of THN by community members will be estimated as proportions. The outcomes will also be estimated for each THN formulation (including the multiple intranasal and injectable formulations that will be captured in this study).

All secondary and exploratory endpoints will be estimated as proportions. Demographic and clinical characteristic data for study participants will be reported using summary statistics for continuous variables (means and standard deviations or medians and interquartile ranges) or frequencies and percentages for categorical variables, as appropriate.

Descriptive data will be reported as numeric counts and percentages with the denominator for reporting percentages appropriate to the specific question.

The extent of missing data at each quantitative study inquiry stage will be tabulated appropriately.

The key population under investigation will be those included in the witnessed overdose cohort (~600). It is anticipated that this will only have missing item level data, as by being in the witnessed overdose cohort, they will be followed up. We do not anticipate any additional analyses in those lost to follow up between those initially enrolled (~6,000) and those included in the witnessed overdose cohort primary population (n ~ 600). We are monitoring the event rate within those initially enrolled as it could be the case, that we could stop enrolment sooner than anticipated, if the event rate is considerably higher than initially estimated.

Planned subgroup analysis will include the following: type of participant (e.g., participant witnessed an opioid overdose event, or experienced an opioid overdose event), age of participant, sex of participant, THN formulation, and carrier of THN versus non-carrier.

This study is purely descriptive in nature. No inferential statistics are planned, but, where appropriate, 95% confidence intervals will be calculated for estimates of means and proportions. The data will be collated within a REDCap database and analysed using Stata.

#### Qualitative data analysis

All qualitative interviews of Sub-study B will be transcribed verbatim, and analyses will follow the principles of Iterative Categorization. All interview transcripts will be entered into the specialist qualitative software programme MAXQDA for systematic line-by-line coding. An initial coding frame will be developed based on the topic guide (deductive codes) and this will be supplemented with inductive codes emerging from the interviews themselves. Once all the data have been coded, data extracts relating to key codes will be exported into Microsoft Word for a two-staged analytical process. First, the coded data will be reviewed descriptively to ascertain themes and patterns in the data (including naloxone understanding and training needs, carriage, use, administration, effectiveness, adverse events etc.). Second, the data will be analysed more interpretatively to explore how the various naloxone products compare; any differences between participant groups; any novel concepts and constructs emerging from the data; and how the findings link back to relevant policies, local practices, existing research evidence, and legal and regulatory frameworks.

Transcripts of qualitative interviews will be assessed by a panel of expert addiction clinicians who will examine the interview data collected (i.e., the recordings of interviews) – on which basis they will provide a scored judgement on three a priori determined clinical areas of interest, accompanied by a brief overall narrative summary. The four clinical areas of interest will be scored using a five-point Likert scale. The areas of interest will be: the accuracy of diagnosis of the probable opioid overdose crisis; the accuracy of identification of respiratory depression; the appropriateness of actions taken (including specifically of any naloxone administered as well as examination of appropriateness or otherwise of instances where naloxone was not administered); and the adequacy of the post-naloxone aftercare.

### Study sample size

Previous studies have found that approximately 10% of those supplied with THN witness an opioid overdose [19–22].

In a cohort of 600 participants who witness an opioid overdose event, we would anticipate between 6 to 63% of these events to occur when there was access (carriage/availability) to THN and for it to be used to reverse an opioid overdose in real-world practice.

#### Co-primary outcome: estimation of the true administration of THN

Therefore, using an asymptotic approach, a minimum of 553 participants would need to be interviewed to estimate the true incidence of THN administration (assumed to be 10%) with a confidence width of ±2.5% [23]. Thus, we estimate that the recruitment of 6,000 participants will lead to approximately 600 witnessed opioid overdose events (our main population for investigation) allowing for an estimation of the administration rate of THN with a precision of ±2.5%.

#### Co-primary outcome: estimation of mortality rate

Assuming a morality rate of 1.4% [20, 22], using a sample size of 600 will allow the estimation of the 95%CI within a width of ±1.5%. We anticipate approximately 8 deaths from the 600 witnessed overdoses reported by participants.

## Discussion

Whilst we know naloxone has the potential to save lives, little is known about its effectiveness in real-life settings when administered by community members to reverse opioid overdoses. This study aims to evaluate THN (in all forms) administered by community members in reversing opioid overdose in real-life situations and settings across Europe.

There are many challenges involved in evaluating the effectiveness of THN due to the complexities involved in studying its use in real-world conditions which can be unpredictable. First, the recruitment of our target population (laypeople provided with THN) will depend upon recruitment sites distributing THN kits. The type of THN Kit received by participants (and administered) will depend on the type of naloxone given out by sites, which will depend upon the type of naloxone marketed, distributed, and used, in countries and local regions as well as the sites’ preference. Second, the study is primarily interested in recruiting and interviewing those community members who have received THN who have witnessed an opioid overdose in the study period. For the primary outcome measures to be meaningful, the study participant will need to have been present at an opioid overdose during the period between enrolment and six months post enrolment. However, it is impossible to predict the exact number or proportion of those provided with THN who will witness an opioid overdose. Third, following up a hard-to-reach population that often experiences instability will be difficult over the 6-month study period. We hope to reduce attrition by staying in contact with participants through regular text message reminders and recruitment site staff contact. Fourth, the study will also incorporate a Post Authorisation Efficacy Study as part of the regulatory requirements for the concentrated naloxone nasal spray, and the cohort design has had to incorporate some additional components to achieve this aim.

### Supplementary Information

**Additional file 1.** NalPORS Questionnaire.

**Additional file 2.** Brief Topic Guide.

## Data Availability

Not applicable.

## Abbreviations

ADR: Non-serious adverse drug reaction
EEA: European Economic Area
IM: Intramuscular
IN: Intranasal
IV: Intravenous
NSP: Needle and syringe programmes
OUD: Opioid use disorder
PAES: Post Authorisation Efficacy Study
PIN: Unique study number
SADR: Serious adverse drug reactions
SC: Subcutaneous
THN: Take home naloxone

## Footnotes

1 Concentrated naloxone nasal-spray formulations and pre-filled syringes of injectable naloxone refer primarily or exclusively to Nyxoid or Prenoxad respectively.
