# Supplementary material for: A study protocol for a European, mixed methods, prospective, cohort study of the effectiveness of naloxone administration by community members, in reversing opioid overdose: NalPORS
Source: BMC Public Health. 2023 Aug 24;23:1608. doi: 10.1186/s12889-023-16445-6 (PMC10463843; doi:10.1186/s12889-023-16445-6)
Supplement: Supplementary file 2 — Additional file 2. Brief Topic Guide. [file 12889_2023_16445_MOESM2_ESM.docx]

**Brief Topic Guide**

| ***Part 1: Participant’s background***   - Demographic information (sex, age, ethnic background, family, social support, housing, income/employment, health) - Current drug use and drug use history - Current treatment and treatment history - Previous experience of overdose (actual and witnessed)   ***Part 2: Overdose intervention (most recent event)***   - Background information to overdose event (location, participants involved and relationships, time of day, drugs used and how used) - Recognising and responding to overdose (factors influencing overdose, recognising overdose, responses to overdose, decisions underlying actions taken) - Role of emergency services in overdose event (if/who called, communication on site with emergency services)   ***Part 3: Use of naloxone***   - Decisions underlying use of naloxone (why, who, and how / type of kit used) - How much naloxone used (dose, number of shots, reasons for variation) - Use of equipment (ease/difficulty, reading/use of instructions, feelings at point of delivery) - Role of bystanders (assisting/preventing action) or intervened alone (problems/benefits) - Immediate response of person who overdosed to naloxone (how long, describe how person reacted on reversal)   ***Part 4: Overdose-reversal management***   - Physical / psychological responses of person who overdosed (how managed, expectations of participant/overdose; use/reuse of opiates or drug seeking behaviour following reversal) - Conflict / emotional management by participant / bystanders - Recognition of withdrawal symptoms (what symptoms, how recognised and how managed) - Participant’s views on administration naloxone after observing reaction - Disposal of naloxone kit/equipment (including whether kit replaced or not)   ***Part 5: Contact with professionals***   - Participant’s experiences of contact with 999 services/other at scene of overdose (problems, benefits, outcome) and also following event - Participant’s (emotional) experiences of witnessing overdose   ***Part 6: Impact of overdose***   - Any participant concerns about the overdose event? - Participant’s views on naloxone use/ drug use following intervention - Preparedness to respond again in further overdose events |
| --- |
